# Development of a broad-lineage Lassa virus rapid diagnostic test informed by the WHO REASSURED framework

**DOI:** 10.1128/jcm.00071-26

**Published:** 2026-03-30

**Authors:** Hwachul Shin, Minji Lee, Myung-Min Choi, Jin-Won Kim, Hwajung Yi, Shannon Whitmer, Leanna Sayyad, Joel Montgomery, John Klena, Yoon-Seok Chung

**Affiliations:** 1 Division of High-risk Pathogen, Korea Disease Control and Prevention Agency (KDCA) 204189, Cheongju, Republic of Korea; 2 Division of Laboratory Diagnosis and Analysis, Gyeongnam Regional Center for Disease Control and Prevention, Busan, Republic of Korea; 3 Viral Special Pathogens Branch, US Centers for Disease Control and Prevention Research Centers 144823 , Atlanta, Georgia, USA; Mayo Clinic Minnesota, Rochester, Minnesota, USA

**Keywords:** Lassa virus, rapid diagnostic test, antigen, antibody, REASSURED

## Abstract

Lassa fever is a high-risk viral disease endemic to West Africa for which rapid and reliable diagnosis is essential for outbreak control. Here, we describe the development of a lateral flow-based rapid diagnostic test (RDT) targeting the Lassa virus nucleoprotein using newly generated monoclonal antibody pairs. The analytical performance of the assay was evaluated using human serum and plasma specimens as well as inactivated Lassa virus strains and compared with a commercially available antigen test. The final RDT (Pair 2; 1P-44-7/9B-28) demonstrated high analytical sensitivity and 100% specificity across 60 human samples and detected multiple genetically diverse Lassa virus strains, with lineage-dependent differences in analytical sensitivity. In direct analytical comparisons using recombinant antigen, the assay was approximately 64-fold more sensitive than the ReLASV Pan-Lassa Antigen Rapid Test. The RDT provides a rapid visual readout, remains stable at ambient temperatures, and is suitable for decentralized testing workflows, features aligned with key aspects of the World Health Organization (WHO) REASSURED framework. These results indicate that the developed RDT offers a robust and broadly reactive platform for Lassa virus antigen detection and has potential utility for surveillance and outbreak response in resource-limited settings.

**IMPORTANCE:**

Lassa fever remains a significant public health concern in West Africa, where diagnostic capacity is often limited in endemic regions. This study describes the development of a broad-lineage Lassa virus rapid diagnostic test informed by the World Health Organization (WHO) REASSURED framework. The assay demonstrates high analytical sensitivity and broad lineage coverage in a field-deployable format, supporting its potential utility for early case detection and decentralized surveillance in endemic settings.

## INTRODUCTION

Lassa fever (LF) is an acute viral hemorrhagic disease endemic to West Africa, causing substantial morbidity, mortality, and socioeconomic disruption in affected regions (1, 2). The causative agent, Lassa virus, belongs to the *Arenaviridae* family and is primarily transmitted to humans through contact with infected rodent reservoirs, most notably the multimammate rat *Mastomys natalensis*, as well as secondary reservoirs, including *Hylomyscus pamfi* and *Mastomys erythroleucus*, or their excreta (3, 4). To date, at least seven LASV lineages (I–VII), including sublineages IV.a and IV.b, have been described, contributing to genetic and antigenic diversity across endemic regions. Secondary human-to-human transmission, particularly in healthcare settings lacking adequate infection control measures, can further amplify outbreaks (5, 6).

Timely and accurate LASV diagnosis is critical for outbreak control and mortality reduction. Traditional diagnostic approaches, including virus isolation, enzyme-linked immunosorbent assay (ELISA), and indirect immunofluorescence assays, require sophisticated laboratory infrastructure and trained personnel, limiting their applicability in resource-limited endemic regions (7, 8) and highlighting the need for reliable, affordable, and field-deployable diagnostic tools.

In West Africa, an estimated 100,000–300,000 Lassa fever cases and approximately 5,000 deaths occur annually (9), with case fatality rates ranging from 1% in the general population to as high as 15–20% among hospitalized patients. This epidemiological burden highlights the urgent need for effective diagnostic tools to reduce transmission and support early intervention strategies (10).

Recent developments in rapid diagnostic tests (RDTs), particularly Pan-Lassa antigen detection assays, offer promising solutions. Field evaluations during recent outbreaks in Nigeria reported high diagnostic sensitivity and specificity across diverse LASV lineages, supporting their potential for widespread field use (11, 12). However, the currently available kits exhibit suboptimal sensitivity for certain LASV strains, and visual interpretation of faint test lines can be challenging under full personal protective equipment (PPE) conditions. This variability in assay performance highlights the need for continued validation and optimization.

Beyond diagnostic advances, elucidating LASV epidemiology and ecology is vital for effective prevention and control. Spatial mapping and ecological studies have delineated high-risk zones, guiding targeted public health interventions (13, 14). Moreover, the discovery of additional reservoir hosts continues to highlight the ecological complexity and dynamic transmission patterns of LASV (15–17).

Recognizing these challenges, the World Health Organization (WHO) has prioritized the development of improved diagnostics, vaccines, and therapeutics in its Lassa Fever Research and Development Roadmap, emphasizing the importance of coordinated global efforts (18). Addressing these priorities—especially developing field-ready diagnostics and expanding ecological knowledge—is essential for reducing LF incidence and mitigating its public health effects.

To guide the development of suitable diagnostic tools for low-resource settings, WHO proposed the REASSURED criteria, an acronym for Real-time connectivity, Ease of specimen collection, Affordable, Sensitive, Specific, User-friendly, Rapid and robust, Equipment-free, and Deliverable to end users (19). These criteria emphasize practicality, robustness, and accessibility in outbreak-prone regions.

In this study, we aimed to address current gaps in the field by developing a lineage-inclusive, lateral flow-based LASV nucleoprotein (NP)-targeting RDT aligned with key aspects of the WHO REASSURED framework using newly generated high-affinity monoclonal antibodies.

## MATERIALS AND METHODS

### Recombinant antigen production

The full-length LASV *NP* gene (National Center for Biotechnology Information [NCBI] accession no. GU481072) was cloned into a baculovirus expression system. The gene was inserted into the BacPAK vector using *Bam*HI and *Sal*I restriction sites and transfected into *Spodoptera frugiperda* (Sf9) cells. Protein expression was performed in serum-containing medium at 27°C. The recombinant NP antigen containing a C-terminal His-tag was purified using Ni-NTA affinity chromatography, followed by SDS–PAGE analysis and bicinchoninic acid assay-based quantification.

### Monoclonal antibody generation

This study was conducted in accordance with institutional and international guidelines for the ethical use of animals in research. All animal experiments were reviewed and approved by the Institutional Animal Care and Use Committee (IACUC) under approval number AEC-20080519-0001. A group of 6-week-old female BALB/c mice was used for monoclonal antibody generation. The mice were housed under specific-pathogen-free conditions, maintained at 22 ± 2°C with 50 ± 10% humidity and a 12-h light/dark cycle, and provided with food and water *ad libitum*. Animal health was monitored daily, and humane endpoints were applied in accordance with institutional policies. This study was designed and conducted in accordance with the ARRIVE 2.0 guidelines (20), aiming to improve animal research transparency and reproducibility.

Six-week-old female BALB/c mice were immunized intraperitoneally with recombinant NP emulsified in complete Freund’s adjuvant. One week later, the mice received a booster immunization with the same antigen emulsified in incomplete Freund’s adjuvant, followed by two additional intraperitoneal booster doses administered at 1-week intervals. One week after the fourth immunization, mice were further boosted by intravenous injection of the same antigen via the tail vein on three consecutive days. Splenocytes were subsequently harvested and fused with SP2/0 myeloma cells using polyethylene glycol. Anti-NP antibody-secreting hybridomas were screened by indirect ELISA using antigen-coated microplates to identify clones specifically reactive to the target antigen. Positive clones were expanded, and antibodies were purified by Protein G affinity chromatography.

### Antibody pair screening and optimization

Candidate antibody pairs were evaluated using lateral flow immunoassay (LFA) formats using gold-conjugated detector and nitrocellulose-immobilized capture antibodies. Screening was performed using recombinant NP-spiked pooled human serum. Analytical sensitivity and specificity were assessed across a dilution range (1 μg/mL to 0.25 ng/mL) using NP-spiked positive samples and randomly selected negative sera obtained through a collaboration with domestic veterinary diagnostic laboratories.

Sample pad buffer compositions were optimized for LFA use by screening 23 formulations. Gold nanoparticle conjugation was performed using ascorbic acid as a reducing agent. Detector antibody concentration was optimized to achieve an optical density of 8.0 at 540 nm.

Signal amplification strategies, including biotin–streptavidin systems, were considered during assay development.

### LFA format and operation

The Lassa virus RDT is a nitrocellulose-based LFA designed for NP antigen detection. Streptavidin was dispensed onto the membrane at a defined test line position at 2.0 mg/mL using an automated dispenser. Capture monoclonal antibody (1P-44-7) was biotinylated by reacting 2 mg of antibody with 27 μL of 10 mM biotin for 30 min at room temperature, followed by desalting using a PD-10 column prior to immobilization via streptavidin–biotin interaction. Detector antibody (9B-28) was conjugated to colloidal gold nanoparticles at a final antibody concentration of 5 μg/mL. After stabilization, the conjugates were pelleted, resuspended in physiological saline, filtered through a 0.45 μm membrane, and diluted to the working concentration. Assay conditions, including conjugation parameters and sample elution volume, were empirically optimized to maximize signal intensity while minimizing background. The optimized LFA strips were subsequently assembled into plastic housings and validated. Upon the application of 80 μL of sample to the sample pad, capillary action drives the sample through the conjugate pad, where the detector antibody binds to the NP antigen. The antigen–antibody complex migrates to the test line and is captured by the immobilized antibody, forming a visible red line within 15 min in the presence of the NP. The test includes a control line for procedural validity and is interpreted visually. All testing was performed at an ambient temperature (20–30°C).

### Epitope mapping and antibody characterization

Epitope mapping was conducted using seven overlapping NP fragments (each ~80–100 amino acids), which were amplified based on the Lassa virus NP gene sequence (NCBI accession no. GU481072), cloned into the *Escherichia coli* expression vector pQE30, and expressed in *E. coli*. The truncated NP fragments (regions 1–7) were purified under denaturing conditions using nickel-affinity chromatography following solubilization in 8 M urea. Nitrocellulose-immobilized monoclonal antibodies were probed with gold-conjugated NP fragments to localize the binding epitopes.

Antibody isotyping was performed using the Pierce Rapid ELISA Mouse mAb Isotyping Kit (Thermo Scientific, Waltham, MA, USA) according to the manufacturer’s instructions. Variable region sequences, including complementarity-determining regions, were obtained from selected monoclonal antibodies using RNA extraction from the corresponding hybridoma cells, followed by cDNA synthesis and PCR amplification (see Table S4).

### RDT kit stability testing

The long-term stability of the developed RDT kits was evaluated for up to 24 months under ambient storage conditions (20–30°C), reflecting typical room-temperature conditions that ensure stable reaction kinetics and reproducible pad performance. Kits were packaged in sealed foil pouches with desiccant and tested every 3 months using recombinant LASV NP antigen to assess analytical sensitivity.

Stability testing was performed using the final RDT configuration (Pair 2; 1P-44-7/9B-28), and LoD was evaluated based on the ≥95% detection criterion. At each time point, the LoD was evaluated using the same ≥95% detection criterion based on replicate testing with recombinant NP.

### Surface plasmon resonance (SPR) analysis

To evaluate the binding affinity between monoclonal antibodies and the NP antigen, SPR measurements were carried using the Biacore T200 system. The recombinant NP antigen was immobilized on a CM5 sensor chip via EDC–NHS coupling chemistry. Kinetic constants (ka, kd, and KD) and steady-state affinities were determined for five monoclonal antibodies using multiconcentration injection protocols. Regeneration was performed using Glycine-HCl (pH 1.5). Data were analyzed using the Biacore evaluation software.

### Analytical performance evaluation

The limit of detection (LoD) was defined as the lowest antigen concentration detected in ≥95% of replicate tests (*n* ≥ 20), determined by binomial probability analysis using serial dilutions of recombinant NP. To evaluate specificity, a total of 60 presumably negative human blood samples that were collected in South Korea, comprising 20 serum, 20 EDTA plasma, and 20 EDTA-sodium citrate plasma samples, were tested.

Cross-reactivity testing included Japanese encephalitis, yellow fever, Zika, dengue, and Ebola viruses, as well as *Plasmodium falciparum* and *Plasmodium vivax*. Japanese encephalitis virus was included as a representative flavivirus commonly present in Asian blood donor populations to evaluate potential off-target reactivity in human specimens. Lujo virus was not included in the analytical specificity panel because inactivated material was not available at the time of this study.

Interference studies were conducted using human anti-mouse antibodies (HAMA), hemoglobin, and bilirubin. Interference testing was performed by spiking recombinant LASV NP into human plasma, followed by the addition of these interferents at sufficiently high concentrations to assess their impact on antigen detection. All tests were performed at 25°C and 40–60% relative humidity.

### Real-time RT-PCR for LASV detection

Lassa virus RNA detection was performed using the Lilif LASV Real-Time RT-PCR Kit (Intronbio, Korea) following the manufacturer’s protocol. Each 20-μL reaction contained 10 μL of 2× RT PCR Mix, 5 μL of LASV detection solution, and 5 μL of sample (positive control, negative control, or extracted RNA). Thermal cycling was performed as follows: reverse transcription at 50°C for 15 min, initial denaturation at 94°C for 5 min, followed by 5 cycles of pre-amplification (94°C for 15 s, 58°C for 30 s) and 35 cycles of amplification (94°C for 15 s, 58°C for 30 s, with fluorescence acquisition at 58°C). Fluorescence channels were configured as FAM and Cy5 for the LASV L and internal process control genes, respectively. No ROX reference dye was used.

### Ethics statement for human samples

The human serum and plasma samples used in this study were residual specimens originally collected for national disease surveillance and control purposes by the Korea Disease Control and Prevention Agency (KDCA). All samples were anonymized and de-identified prior to use in this study; therefore, the requirement for Institutional Review Board (IRB) approval was waived.

## RESULTS

### Antibody pair and signal enhancement optimization

To identify an optimal antibody combination for LASV antigen detection, multiple antibody pairs were screened using LFAs. The antibody pair developed in prototype 2018 (capture: 1P-44-7; detector: 1P-88-3) was excluded due to false-positive signals (data not shown). An alternative pair (Pair 1; capture: 1P-44-7; detector: 1P-39-1) exhibited markedly improved sensitivity, achieving an LoD of 0.98 ng/mL, representing an ~8 fold improvement over the original pair along with improved specificity (93%) (Table S2). Further screening identified two additional candidate pairs: pairs 2 (capture: 1P-44-7; detector: 9B-28) and 3 (capture: 10F-54; detector: 1P-39-1). Despite displaying the lowest LoD (0.25 ng/mL), Pair 3 yielded only 40% specificity when tested against 60 negative serum/plasma samples (Table S2). In contrast, Pair 2 maintained 100% specificity with a satisfactory LoD of 0.98 ng/mL. Based on the optimal sensitivity and specificity balance, Pair 2 (1P-44-7/9B-28) was selected for incorporation into the final RDT kit.

To enhance sensitivity, a biotin–streptavidin amplification system was also evaluated using three candidate antibody pairs. This approach reduced the LoD to 0.49 ng/mL, representing an improvement of up to 16-fold over the 2018 prototype. However, weak nonspecific signals were observed in several negative samples, particularly for 10F-54–biotin combinations (Table S3). Therefore, the amplification strategy was not included in the final assay design.

### Analytical specificity and cross-reactivity assessment

The experiments were conducted exclusively with the final candidate antibody pair (1P-44-7/9B-28). No cross-reactivity was observed with Japanese encephalitis, yellow fever, Zika, dengue, or Ebola (NP, GP, and VP40) viruses, nor with *Plasmodium* spp. (malaria). No false-negative or -positive results were observed when hemoglobin (10–25 g/L), bilirubin (>400 mg/dL), or HAMA (titer 69-802) was added to NP-spiked human plasma, indicating that these common interferents did not compromise assay performance.

Further assessment was conducted using other hemorrhagic fever viruses, including five Ebola virus subtypes, two Marburg virus subtypes (Marburg and Ravn), as well as Crimean-Congo hemorrhagic fever, Rift Valley fever, and lymphocytic choriomeningitis viruses (CCHFV, RVFV, and LCMV, respectively). All samples tested negative at a 1:10 dilution, confirming the absence of cross-reactivity with the tested viral pathogens (Table 1).

**TABLE 1:** Analytical specificity of the final Lassa virus RDT^*a*^

Pathogen	Family	Form tested	Matrix	Result
Zaire ebolavirus	Filoviridae	Inactivated virus	Human plasma	Not detected
Sudan ebolavirus	Filoviridae	Inactivated virus	Human plasma	Not detected
Bundibugyo ebolavirus	Filoviridae	Inactivated virus	Human plasma	Not detected
Taï Forest ebolavirus	Filoviridae	Inactivated virus	Human plasma	Not detected
Reston ebolavirus	Filoviridae	Inactivated virus	Human plasma	Not detected
Marburg virus	Filoviridae	Inactivated virus	Human plasma	Not detected
Ravn virus	Filoviridae	Inactivated virus	Human plasma	Not detected
Crimean-Congo hemorrhagic fever virus	Nairoviridae	Inactivated virus	Human plasma	Not detected
Rift Valley fever virus	Phenuiviridae	Inactivated virus	Human plasma	Not detected
Lymphocytic choriomeningitis virus	Arenaviridae	Inactivated virus	Human plasma	Not detected
Plasmodium falciparum	Protozoa	Whole parasite lysate	Human plasma	Not detected
Plasmodium vivax	Protozoa	Whole parasite lysate	Human plasma	Not detected
Lassa virus (Josiah)	Arenaviridae	Inactivated virus	Human plasma	Detected

^*a*^ Cross-reactivity was evaluated using inactivated viral preparations and blood-borne pathogens spiked into human plasma. All tests were performed using the final RDT (Pair 2; 1P-44-7/9B-28). Pathogens were tested at high challenge concentrations (≥10⁵ *TCID*_50_/mL for viruses or equivalent antigen levels).

### Comparative performance and antibody binding characteristics

The performance of the developed RDT was benchmarked against a commercial kit (Zalgen, Germantown, Maryland, USA) using the recombinant NP antigen. At 62.5 ng/mL, the commercial kit yielded a weak signal (≤3%), whereas the newly developed RDT (Pair 2) generated approximately 50% signal intensity. The new assay consistently detected antigen concentrations as low as 0.98 ng/mL, demonstrating approximately 64-fold higher sensitivity than that of the comparator (Table S1).

SPR analysis confirmed that the selected antibody pair, 1P-44-7 (capture) and 9B-28 (detector), exhibited high binding affinity (KD < 1 × 10^−7^ M), stable response units (RU), and acceptable Chi² values. These results were consistent with the LFA performance data and supported the selection of this pair for the final RDT (Fig. S1).

### Epitope mapping confirmed pair compatibility

**Fig 1:**
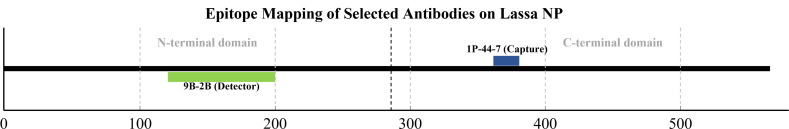
Epitope mapping of selected monoclonal antibodies using truncated Lassa virus nucleoprotein fragments. Overlapping Lassa virus nucleoprotein fragments were expressed in *Escherichia coli*, then purified and probed with gold-conjugated monoclonal antibodies. The capture (1P-44-7) and detector (9B-28) antibody binding regions were mapped to distinct, nonoverlapping domains. Figure keys: NP, nucleoprotein; aa, amino acids.

Epitope mapping using truncated NP fragments confirmed the absence of overlapping binding sites for the selected antibodies. The capture antibody 1P-44-7 recognized the N-terminal region (amino acids 361–380), whereas the detector antibody 9B-28 bound to the C-terminal region (amino acids 121–200). These distinct binding sites validated the compatibility of this pair for use in sandwich-format LFAs (Fig. 1).

### Validation using inactivated Lassa virus strains

The analytical performance of the final RDT (Pair 2; 1P-44-7/9B-28) was evaluated using inactivated Lassa virus strains representing multiple genetic lineages in collaboration with the Viral Special Pathogens Branch of the US Centers for Disease Control and Prevention. Serial dilutions (1:5 to 1:160) of each inactivated virus preparation were tested to determine the dilution endpoints of antigen detection.

The final RDT exhibited the highest sensitivity for the Josiah strain, yielding positive results up to a 1:80 dilution. For the other strains, the detection endpoints were as follows: 1:20 for the Togo strain, 1:10 for NJ2015, 1:20 for SLE Niahun, and 1:10 for the NGR strain (Table 2). These results demonstrate that the assay is capable of detecting Lassa virus antigens across genetically diverse strains, although lineage-dependent differences in analytical sensitivity were observed.

**TABLE 2:** Detection endpoints of the final RDT across five inactivated Lassa virus strains^*b*^

Strain	Lineage^*a*^	1/5	1/10	1/20	1/40	1/80	1/160	Detection endpoint	Starting Ct values
Josiah	IV	+	+	+	+	+	−	1:80	17.72
Togo	II	+	+	+	+	−	−	1:20	24.86
NJ2015	VI	+	+	−	−	−	−	1:10	21.21
SLE Niahum	IV	+	+	+	+	−	−	1:20	19.64
NGR	III	+	−	−	−	−	−	1:10	25.90

^*a*^ Lineage assignments should be specified based on CDC characterization.

^*b*^ Serial dilutions (1:5–1:160) of inactivated Lassa virus strains were tested using the final RDT (Pair 2; 1P-44-7/9B-28). Detection endpoints indicate the highest dilution at which the test line was observed in ≥95% of replicate tests.

### Long-term RDT kit stability

Stability testing demonstrated that the final RDT (Pair 2; 1P-44-7/9B-28) maintained its original LoD of 0.98 ng/mL for at least 18 months under ambient storage conditions (20–30°C). After 21 months, a moderate loss of sensitivity was observed, with the LoD increasing to 1.96 ng/mL, while control line performance remained unaffected.

## DISCUSSION

In this study, we developed and validated a lateral flow-based RDT for LASV antigen detection using newly generated monoclonal antibodies. The selected antibody pair (1P-44-7/9B-28) demonstrated high specificity and sensitivity, forming the basis for subsequent validation across LASV strains.

The selected antibody pair demonstrated high binding affinity (KD < 1 × 10^−7^ M) and recognized nonoverlapping epitopes, making it well-suited for sandwich immunoassay configurations. Validation using inactivated viral strains confirmed reliable detection of the Josiah strain at diagnostically relevant titers, yielding positive results up to a 1:80 dilution. The RDT also successfully detected diverse LASV lineages, including strains from Togo, NJ2015, NGR, and SLE Niahun, thereby indicating broad clade coverage.

Importantly, the assay revealed no cross-reactivity with other hemorrhagic fever viruses, nor with lymphocytic choriomeningitis virus (LCMV), a closely related arenavirus that causes early clinical symptoms highly similar to Lassa fever. In addition, assay performance was unaffected by common interferents, such as hemoglobin, bilirubin, and HAMA. Collectively, these findings demonstrate the high analytical specificity and potential field utility of this assay (Table 1; Tables S2 and S3).

Compared with the currently available ReLASV Pan-Lassa Antigen Rapid Test (Zalgen), which has shown reduced visual interpretability under full personal protective equipment (PPE) conditions in previous field studies (14), our RDT demonstrated an 8–32-fold improvement in analytical sensitivity under controlled laboratory conditions (Table S1).

The assay aligns with the WHO REASSURED framework and is compatible with reader-based connectivity approaches (19). Supporting features include stability at ambient temperatures, visual interpretation within 15 min, and pan-lineage detection capability. A summary of REASSURED compliance is provided in Table S6.

Nevertheless, the study was limited by the absence of clinical validation using acute-phase patient samples. This limitation might result in an overestimation of the diagnostic performance of the test under idealized laboratory conditions. Future research should prioritize field evaluations in endemic settings to confirm diagnostic performance under real-world conditions.

Overall, these results demonstrated the diagnostic utility and operational feasibility of the developed RDT, supporting its integration into Lassa fever surveillance and outbreak response systems. The suitability of this assay for decentralized use highlights its potential applicability in early case detection and LF outbreak containment in endemic regions.

## Data Availability

All data supporting the findings of this study are included within the article and its supplementary materials. Additional information is available from the corresponding author upon reasonable request.

## SUPPLEMENTAL MATERIAL

Figure S1 jcm.00071-26-s0001.tif Sensorgrams of monoclonal antibodies 1P-44-7 (gray) and 9B-28 (deep blue) binding to immobilized LASV nucleoprotein on a CM5 chip. *K*_*D*_ values (log scale) were determined using Biacore T200. RU, response unit; *K*_*D*_, equilibrium dissociation constant.

Figure S2 jcm.00071-26-s0002.tif Manufacturer-provided reference color scale used for visual interpretation of test line intensity in the Lassa virus rapid diagnostic test.

Supplemental result jcm.00071-26-s0003.docx Supplemental result.

ASM does not own the copyrights to Supplemental Material that may be linked to, or accessed through, an article. The authors have granted ASM a non-exclusive, world-wide license to publish the Supplemental Material files. Please contact the corresponding author directly for reuse.
