# Supplementary figures and images for: Development of a broad-lineage Lassa virus rapid diagnostic test informed by the WHO REASSURED framework
Source: J Clin Microbiol. 2026 Mar 30;64(5):e00071-26. doi: 10.1128/jcm.00071-26 (PMC13170227; doi:10.1128/jcm.00071-26)

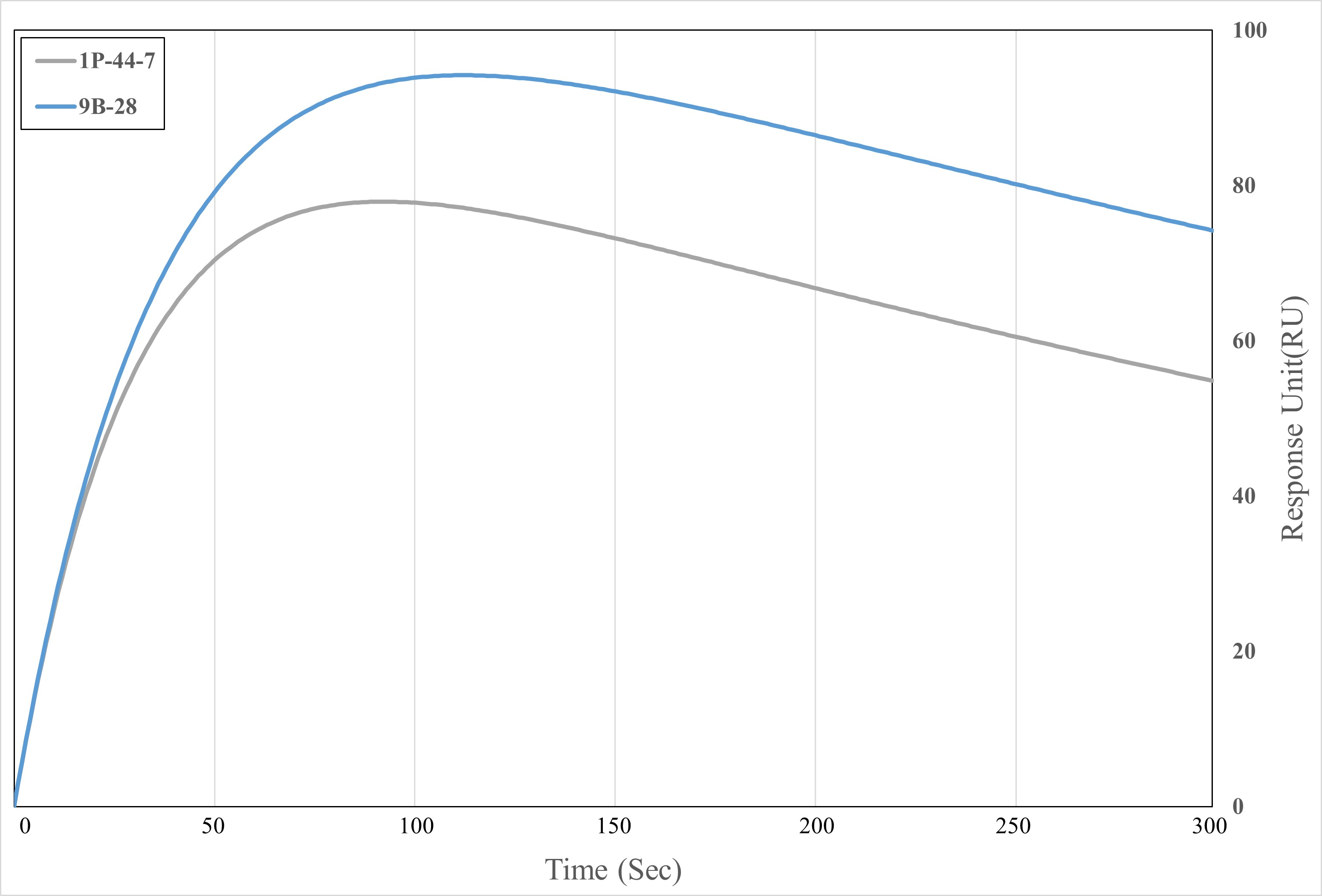

Supplement: Figure S1 — Sensorgrams of monoclonal antibodies 1P-44-7 (gray) and 9B-28 (deep blue) binding to immobilized LASV nucleoprotein on a CM5 chip. KD values (log scale) were determined using Biacore T200. RU, response unit; KD, equilibrium dissociation constant. [file jcm.00071-26-s0001.tif]

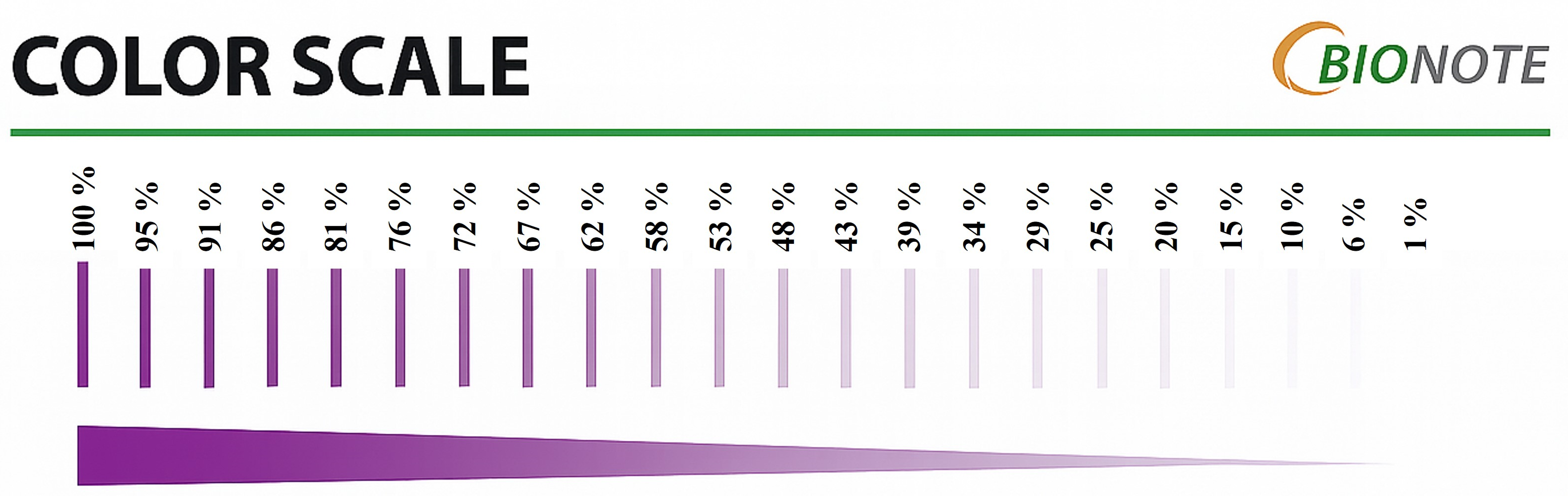

Supplement: Figure S2 — Manufacturer-provided reference color scale used for visual interpretation of test line intensity in the Lassa virus rapid diagnostic test. [file jcm.00071-26-s0002.tif]
