# Supplementary material for: Development of a broad-lineage Lassa virus rapid diagnostic test informed by the WHO REASSURED framework
Source: J Clin Microbiol. 2026 Mar 30;64(5):e00071-26. doi: 10.1128/jcm.00071-26 (PMC13170227; doi:10.1128/jcm.00071-26)
Supplement: Supplemental result [file jcm.00071-26-s0003.docx]

**Supplementary Table 1.** Signal intensity of candidate antibody pairs across serial Lassa virus antigen dilutions

| # Pair | Capture |  |  | 1P-44 | Streptavidin | |
| --- | --- | --- | --- | --- | --- | --- |
|  | Gold-Ab |  | 1P-88 | 1P-39-1 -gold | 9B-28 -gold | 1P-39-1 -gold |
|  | Biotin-Ab |  | N/A | 1P-44-7 -biotin | 1P-44-7 -biotin | 10F-54 -biotin |
| Antigen concentration (ng/mL) | | Zalgen® Pan-Lassa Antigen Rapid Test | 2018 Prototype  (%*) | Pair 1  (%) | Pair 2  (%) | Pair 3  (%) |
| 1000 | | 29 | 86 | 100 | 100 | 100 |
| 500 | | 20 | 81 | 100 | 100 | 100 |
| 250 | | 25 | 58 | 81 | 72 | 86 |
| 125 | | 6 | 48 | 62 | 72 | 81 |
| 62.5 | | 1 | 29 | 53 | 48 | 62 |
| 31.25 | | 0 | 15 | 29 | 39 | 48 |
| 15.6 | | 0 | 6 | 25 | 20 | 39 |
| 7.8 | | 0 | 1 | 20 | 15 | 29 |
| 3.9 | | 0 | 0 | 10 | 6 | 25 |
| 1.95 | | 0 | 0 | 6 | 6 | 10 |
| 0.98 | | 0 | 0 | 1 | 1 | 6 |
| 0.49 | | 0 | 0 | 0 | 0 | 1 |
| 0.25 | | 0 | 0 | 0 | 0 | 1 |
|  | * Line intensity values correspond to the manufacturer-provided 22-step color scale (1 % to 100 %) shown in Supplementary Figure 1, used for visual scoring of test line signals. | | | | | |

**Supplementary Table 2**. Specificity comparison of antibody pairs using human serum and plasma samples

|  | Serum | Plasma | | Result | |
| --- | --- | --- | --- | --- | --- |
|  |  | EDTA | Sodium citrate |  |  |
| Pair 1  1P-44-7 / 1P39-1  (%) | 1/20 | 1/20 | 2/20 | 4/60 | 93 % |
| Pair 2  1P-44-7 / 9B-28  (%) | 0/20 | 0/20 | 0/20 | 0/60 | 100 % |
| Pair 3  10F-54 / 1P39-1  (%) | 16/20 | 14/20 | 6/20 | 36/60 | 40 % |

**Supplementary Table 3**. Non-specific signal responses in negative serum samples using candidate antibody pairs with a biotin–streptavidin system

| Capture | Streptavidin | Streptavidin | Streptavidin |
| --- | --- | --- | --- |
| Gold-Ab | 1P-39-1 -gold | 9B-28 -gold | 1P-39-1 -gold |
| Biotin-Ab | 1P-44-7 -biotin | 1P-44-7 -biotin | 10F-54 -biotin |
| Negative Sample | Pair 1 | Pair 2 | Pair 3 |
| 6032 | - | - | - |
| 6033 | - | - | - |
| 6034 | - | - | - |
| 6035 | 1 %* | - | 1 % |
| 6037 | - | - | - |
| 6039 | - | - | - |
| 6040 | - | - | - |
| 6041 | 1 % | - | - |
| 6042 | 1 % | - | - |
| 6043 | - | - | - |
| 6044 | 1 % | - | - |
| 6045 | - | - | - |
| 6047 | - | - | - |
| 6048 | - | - | 1 % |
| 6050 | - | - | - |
| 6054 | - | - | - |
| 6052 | - | - | - |
| 6105 | - | - | - |
| * Line intensity values correspond to the manufacturer-provided 22-step color scale (1 % to 100 %) shown in Supplementary Figure 1, used for visual scoring of test line signals. | | | |

**Supplementary Table 4**. Complementarity-determining region sequences of selected monoclonal antibodies

| Antibody | Chain | CDR1 | CDR2 | CDR3 |
| --- | --- | --- | --- | --- |
| 1P-44-7 | VH | GFTFSSFGMH | YISSGSSTLHY | ARQVLHYFDY |
|  | VL | KSSQSLLNSRTRKNYLA | WASTR | KQSYDLPT |
| 9B-28 | VH | GFTFNTYG | IRSKSNNYAT | VRQSSFFYGYAMDC |
|  | VL | RASKSVSTSGYSYM | LVSNLES | QHIRELTR |

**Supplementary Table 5**. Long-term stability of the developed Lassa virus rapid diagnostic test kits under ambient storage conditions

| Time point (months) | Limit of detection (ng/mL) | Control line validity | Functional failure observed |
| --- | --- | --- | --- |
| 3 | 0.98 | Valid | No |
| 6 | 0.98 | Valid | No |
| 9 | 0.98 | Valid | No |
| 12 | 0.98 | Valid | No |
| 15 | 0.98 | Valid | No |
| 18 | 0.98 | Valid | No |
| 21 | 1.95 | Valid | No |
| 24 | 1.95 | Valid | No |

**Supplementary Table 6**. Evaluation of the developed Lassa virus rapid diagnostic test according to WHO REASSURED criteria

|  | **Definition** | **Evaluation for the developed Lassa virus rapid diagnostic test** |
| --- | --- | --- |
| **Real-time** | Provides rapid results at point-of-care | Results available within 15 min |
| **Easy to use** | Requires minimal training or specialized skills | Simple visual interpretation, no complex procedures |
| **Affordable** | Cost-effective for resource-limited settings | Designed for low-cost production using standard LFA materials |
| **Sensitive** | Reliably detects low pathogen levels | LoD = 0.98 ng/mL, detects Ct 17–18 equivalent viral load |
| **Specific** | High accuracy with minimal false positives | 100% specificity across serum and plasma matrices (see Supplementary Table 2) |
| **User-friendly** | Intuitive handling for non-laboratory users | Integrated test strip; no reader or mixing steps required |
| **Rapid** | Delivers timely results during outbreak | Total turnaround time < 15 min |
| **Equipment-free** | Does not require additional devices or electricity | Fully manual, suitable for field and rural conditions |
| **Deliverable** | Easily distributable and field-deployable | Room temperature stable for 12 months, compatible with existing supply chains |

**Supplementary Figure 1. Surface plasmon resonance analysis of antibody binding kinetics to Lassa virus nucleoprotein antigen**

Sensorgrams show binding responses of monoclonal antibodies at multiple concentrations to immobilized nucleoprotein on a CM5 chip. Binding kinetics were analyzed using a Biacore T200 system, and equilibrium dissociation constants (K<sub>D</sub>) are shown on a logarithmic scale. Sensorgrams for antibodies 1P-44-7 (grey) and 9B-28 (deep blue) are indicated.
Figure Keys: RU, response unit; KD, equilibrium dissociation constant

**Supplementary Figure 2.** Manufacturer-provided color scale for the visual interpretation of test line intensity

The figure shows the reference color scale provided by the manufacturer (BIONOTE) for interpreting the visual intensity of the test lines in the developed Lassa virus rapid diagnostic test. The scale is divided into 22 gradations, corresponding to signal reactivity levels from 1 % (lowest) to 100 % (highest). This standardized color reference was used to visually score the test line intensities during analytical sensitivity and stability assessments.
